# The Future of British Nursing


**Published:** 1916-09-16

**Authors:** 


### THE FUTURE OF BRITISH NURSING.*

### III.—TRAINING AND CERTIFICATION.

In the first article we intimated that there was an economic side to this question; that is to say, that no voluntary hospital is justified in spending the money subscribed to it for the relief of the sick and injured poor upon the training of a larger number of nurses than that actually required for the service of the sick in the hospital to which the training-school is attached. The cost—that is to say, the actual cost of each in-patient—has increased by leaps and bounds in the last two decades, and the tendency where science comes in is always to increase, no matter how careful and thorough may be the checks upon expenditure and the ability brought to bear upon the administration of each department of a great hospital.

After the war we must therefore be prepared to find that when the increased demand for trained nurses makes itself insistently manifest, other means than those at present existing and provided by the British nurse-training schools will have to be forthcoming, in which case we foresee that such additional means of training may arise, out of a reconstruction and co-ordination of the whole hospital system of Great Britain, upon lines which will meet the requirements of all classes of the population by providing the means whereby everybody, high or low, who has to undergo a serious operation or to suffer the risks of an acute and dangerous illness, may find the accommodation they need in a hospital or kindred establishment. Here everything that science, knowledge, and medical skill can provide will be always available for the use of all the patients, quite irrespective of the class of the population from which they may come.

If this view has substance, as we think it has, in any case an opportunity will come for the wise modification of the habit of trying to enforce a rule that before any nurse is granted her certificate she shall have completed the whole of her three years' training under the same matron. Directly our hospital system is extended, reconstructed and made adequate to meet the requirements of all classes of the population, the facilities for the better, fuller, and completer training of every nurse will become a relatively simple matter. The standard of hospital treatment will be high everywhere—that is, it will be as good as it is possible to make it. In such circumstances a nurse in the course of her training will have probably facilities for seeing a

* Previous articles appeared in The Hospital of August 12, p. 455, and September 9, p. 545.

variety of methods of doing the work of a nurse, especially where she is ambitious to become fully equipped and to get a knowledge of most, if not all, the many fields into which nursing is necessarily divided.

We have tried in these articles to lead our readers to open the windows of their mind, to get a broader and wider view of the facilities provided for probationers in the years of their training, and to encourage thoughts which will bring fresh minds and high ideals to the task of providing, through the College of Nursing, the widest possible basis on which to establish a complete system of training for British nurses. The ideal system should avoid narrowness and be capable of securing the most fruitful methods of instruction and the widest and best results for good, not only to the individual nurse, but to the whole profession of nursing.

